# Surgery, Liver Directed Therapy and Peptide Receptor Radionuclide Therapy for Pancreatic Neuroendocrine Tumor Liver Metastases

**DOI:** 10.3390/cancers14205103

**Published:** 2022-10-18

**Authors:** Rejoice Ngongoni, Brendan Visser

**Affiliations:** Department of Surgery, Stanford University School of Medicine, Stanford, CA 94305, USA

**Keywords:** cytoreductive surgery, PRRT, embolization, chemoembolization, radioembolization, liver directed therapy, pancreatic, neuroendocrine tumor, liver metastases

## Abstract

**Simple Summary:**

Pancreatic neuroendocrine tumors are tumors with varying degrees of aggressiveness. The most frequent site of metastasis is the liver. Treatment methods for pancreatic neuroendocrine tumor liver metastases (NETLM) range from medications to surgical resection. The aim of this article is to review the published literature on treatment of pancreatic NETLM using surgery, liver directed therapy (bland embolization, chemoembolization and radioembolization) and peptide receptor radionuclide therapy (PRRT). Surgical resection for patients with resectable disease is associated with the longest survival. Locoregional therapy and PRRT were once reserved for unresectable patients but are now used in increasingly creative ways in combination with surgery to improve symptoms and prolong survival.

**Abstract:**

Pancreatic neuroendocrine tumors (PNETs) are described by the World Health Organization (WHO) classification by grade (1–3) and degree of differentiation. Grade 1 and 2; well differentiated PNETs are often characterized as relatively “indolent” tumors for which locoregional therapies have been shown to be effective for palliation of symptom control and prolongation of survival even in the setting of advanced disease. The treatment of liver metastases includes surgical and non-surgical modalities with varying degrees of invasiveness; efficacy; and risk. Most of these modalities have not been prospectively compared. This paper reviews literature that has been published on treatment of pancreatic neuroendocrine liver metastases using surgery; liver directed embolization and peptide receptor radionuclide therapy (PRRT). Surgery is associated with the longest survival in patients with resectable disease burden. Liver-directed (hepatic artery) therapies can sometimes convert patients with borderline disease into candidates for surgery. Among the three embolization modalities; the preponderance of data suggests chemoembolization offers superior radiographic response compared to bland embolization and radioembolization; but all have similar survival. PRRT was initially approved as salvage therapy in patients with advanced disease that was not amenable to resection or embolization; though the role of PRRT is evolving rapidly

## 1. Introduction

Pancreatic neuroendocrine tumors (PNETs) are a rare group of heterogeneous tumors arising from neuroendocrine cells that are distributed throughout the pancreas. They comprise 1–2% of all pancreatic neoplasms [1] and have a reported incidence of 4.4–4.8 cases per 1,000,000 people with a slight male preponderance [2,3]. Neuroendocrine tumors (NETs) are indolent neoplasms known to metastasize to lymph nodes and the liver. Neuroendocrine tumor liver metastases (NETLM) are a negative predictor of survival in patients [4]. They are present in 15–80% of cases and can be synchronous in 59% of cases [5,6]. Management of liver metastases is dependent on burden of disease, progression on surveillance imaging, and symptom control and requires consideration of surgical, ablative, and systemic therapies.

NETs vary from benign tumors to aggressive carcinomas and are classified histologically by tumor grade and differentiation. Differentiation is categorized into well and poorly differentiated. Grade is determined by mitotic index and Ki67 proliferative index. Grade one (G1) tumors have a mitotic index < 2 or a Ki67 index < 3. Grade 2 (G2) tumors have a mitotic index between 2 and 20 or a Ki67 index from 3 to 20. Grade 3 (G3) tumors have a mitotic index or Ki67 index > 20. The higher index is used to classify grade if there is a discrepancy between the mitotic and Ki67 indices [7]. Prognostic factors of overall survival for NET which have been identified include age at diagnosis, tumor grade, and primary tumor site [6]. Treatment of NETLM is based on a variety of these prognostic factors such as tumor grade and primary tumor site, and metastatic pattern (location, number of metastases, size). Neuroendocrine carcinomas (NEC) are poorly differentiated neoplasms of high grade and are categorized as small cell or large cell NEC [8]. Although surgical management for well differentiated G3 tumors and NEC has recently been shown to improve median overall survival, 35.9 months for G3 PNET vs. 11.3 months for pancreatic NEC [9,10,11], these distinct subsets of tumors are generally treated with chemotherapy because of aggressive tumor biology and are typically excluded from series that look at well differentiated G1/G2 tumors which are discussed in this review.

NETs are indolent tumors, therefore, various locoregional therapies can be used to control symptoms, delay disease progression, and prolong disease survival. The modalities that are used to control disease range from somatostatin analogues to surgical interventions and include, chemotherapy, peptide receptor radionuclide therapy (PRRT), external beam radiation, liver directed embolotherapy, and surgery. There is a spectrum of efficacy which generally increases with invasiveness of treatment method. Surgery (including transplantation) has the highest efficacy but is also associated with the highest risk. Unfortunately, most of these treatments have not been prospectively compared. This review will focus on management of pancreatic NETLM using surgery, PRRT, bland embolization, chemoembolization and radioembolization, knowing that many patients over the course of their disease will probably receive most of these therapies. Providers typically strategize to minimize interactions of the treatments while maximizing prolonging life. Due to the rarity of the disease, please note that most studies are comprised of heterogeneous study populations and include patients with NET of non-pancreatic origin.

In the setting of metastatic disease, decisions about surgical management of metastases must also be made while acknowledging the inherent risk of surgical resection of the primary tumor, e.g., a pancreatic primary tumor with vascular invasion and liver metastases might be approached differently from a small pancreatic tail tumor with no vascular invasion or a small ileal tumor with a metastatic focus in the liver. However, this discussion would be beyond the scope of this review which focuses on management of liver metastases of pancreatic neuroendocrine tumors.

## 2. Surgery

### 2.1. Surgical Resection

Cytoreduction is a powerful treatment tool for neuroendocrine liver metastases that is used to slow the pace of disease by decreasing the net tumor burden. It was initially used for symptom control which was best achieved with ≥95% debulking of tumor [12]. However, cytoreduction via hepatectomy has since been shown to be associated with significant long term five-year overall survival (OS) ranging from 61–88% (Table 1). One of the earliest and largest series with a cohort of 170 patients showed that patients who underwent hepatectomy had a median OS of 81 months, five-year OS of 61% and a low mortality of 1.2% [13]. This was later replicated by Mayo et al. who also compared surgical resection to embolization in 753 patients and showed that resection was associated with a higher median OS and five-year OS of 123 months and 74% compared to 34 months and 30%, respectively [14].

The question of extent of tumor debulking arose in the 1980s when symptom control was achieved in patients who had undergone more than 90% debulking [37]. It was previously thought that removing >90% tumor would be associated with improved survival outcomes and symptom control [13,15]. However, a retrospective study which included 108 patients with gastroenteropancreatic neuroendocrine tumors (GEPNETs) showed that the five-year OS was not different between groups that underwent >70% vs. >90% debulking [31]. The notion of performing a 70% debulking has been confirmed in other studies and is probably significant in patients with numerous metastatic lesions (greater than 10 lesions) where 95% debulking might not be feasible [34]. If debulking 70% of tumor burden offers similar survival benefit in patients with numerous metastatic lesions as higher debulking, then this lower debulking percentage could be tolerated especially if surgery could be combined with other therapies such as embolization and PRRT. However, for patients in whom it is feasible, removal of all grossly visible tumor should still be attempted as it allows for resetting the clock and provides the greatest benefit.

Although it has been established that primary NET resection prolongs survival [14,15,16,17,18], it is debated whether resection of primary tumor in patients with metastatic disease is beneficial. There is concern that resection of the primary tumor in patients with metastatic disease (that is not amenable to resection) results in a pro-inflammatory state that can contribute to growth of the residual disease [38]. On the other hand, removal of the primary tumor might change the tumor biology by reversing tumor-induced immunosuppression [39]. A large cohort study using the National Cancer Database found among 6088 patients with Stage IV PNETs that those who underwent primary tumor resection had a significantly higher median OS of 63.6 months than the unresected group (14.2 months). Young patients with low grade tumors also had improved survival [35]. Series of retrospective studies have supported the former argument and shown that resection of the primary tumor prolongs survival in patients with metastatic NET [17,33]. There is also interest in reducing the burden of disease because removal of the primary tumor might help to increase tumor sensitivity to PRRT [40]. In addition, resection of bulky primary tumor even in the setting of metastatic disease could make a patient amenable to liver directed therapies by reducing the number of fronts on which the battle is fought to delay progression of disease. Although surgery is the most invasive therapy tool associated with a high morbidity (14–27%), this is generally considered acceptable because it confers a significantly larger survival advantage [17,22,24,25,26,27,28,29]. Additionally, most of the complications are Clavien-Dindo grade 1 and 2.

### 2.2. Liver Transplantation

Liver transplantation is an alternative treatment that is rarely used for the management of NETLM. Five-year survival has improved from 49–58% [41,42,43], in earlier studies, to 67–97% in recent studies [44,45,46,47]. Comparison of transplantation and resection has shown higher ten-year survival of 89% to 93% in transplanted patients versus 22% to 75% in resected patients [44,47]. This increase in survival is likely multifactorial including an improvement in patient selection for transplantation. Unfortunately, liver transplantation is not available for most patients due to limited availability of organs. Therefore, it is reserved for select patients who are most likely to benefit from transplantation: patients with low hepatic tumor burden (≤50%), low grade (G1/G2) tumor, unresectable liver metastases, stable disease prior to transplantation, resected primary tumor, and primary tumors that drain via the portal system. Liver transplantation has also been performed in patients who need rescue from hepatic insufficiency after liver resection.

## 3. Liver Directed Therapy

Liver directed therapy consists of various minimally invasive treatment methods that are used to manage NETLM in patients with borderline resectable or unresectable lesions. Since NETLM are preferentially supplied by arterial (80–90%) rather than portal venous blood supply [48], hepatic artery inflow can be preferentially occluded resulting in tumor ischemia and necrosis. Selective embolotherapy, which is performed by targeting segmental or subsegmental arteries that supply the tumor, is generally preferred to preserve as much functional liver parenchyma as possible.

Three embolotherapies will be discussed: transarterial embolization (TAE), transarterial chemoembolization (TACE) and transarterial radioembolization (TARE). Bland embolization, also known as TAE, involves arterial occlusion using embolic agents such as polyvinyl alcohol, gel foam particles, cyanoacrylate, and microspheres [31,32,33,34]. In conventional TACE (cTACE or TACE), high dose chemotherapy is initially administered to liver tumors followed by embolic agents. Chemotherapeutic agents that have been used against NETLM include doxorubicin, streptozocin, cisplatin and mitomycin-C [35,36,37]. Another form of TACE involves administration of drug-eluting beads (DEB-TACE). This is similar to TARE where radioactive ^90^Yittrium microspheres are selectively administered intra-arterially to deliver high doses of radiation within a certain radius surrounding the microspheres, resulting in tumor necrosis. In both TACE and TARE, embolization of arteries after administration of agent decreases the dilution of intra-hepatic chemotherapy and increases dwell times and subsequent tumor necrosis [49]. These methods could be used alone or combined with systemic therapy to achieve locoregional control of advanced or metastatic disease.

### 3.1. Transarterial Embolization and Chemoembolization

TAE/TACE are liver directed therapies with multiple indications. Patients selected for embolization typically have borderline resectable or unresectable NETLM or are poor surgical candidates despite having resectable tumors. Low residual functional liver parenchyma and insufficient portal inflow to the liver are associated with poor outcomes making them contraindications to embolization. Other contraindications include hepatic encephalopathy and biliary obstruction due to increased risk for biliary necrosis [49]. Patients who benefit from TACE are those with low liver tumor burden, i.e., <50%, as they have higher lipiodol uptake [50] and higher median PFS and OS [51].

TAE and TACE are associated with improved outcomes. As illustrated in Table 2, both methods have complete and partial imaging response rate to therapy of 35–82% [52,53]. They decrease tumor burden, improve symptoms, and increase survival [32,34,35,37,41,42,43,44,54]. The addition of chemotherapy to TAE was expected to improve response to therapy and outcomes of bland embolization yet studies have been equivocal. Although the median OS in single arm TACE studies is reportedly higher (33–65 months) [53,55,56] than that of single arm TAE studies (24–36 months) [19,57], combined TAE/TACE studies have reported median OS of 23–44 months [14,52,58,59,60] that is comparable to single-arm TACE studies, with a five-year survival of 13–44% [58,60]. Unfortunately, comparative retrospective studies have provided conflicting data. One study reported similar median OS of 25 months in TAE and TACE arms [58] while another study with 84 patients observed that patients in the TACE group had a lower median OS of 44 months compared to 48 months in TAE [57]. In the latter study, the TACE group had a higher number of PNET tumors which portend a poorer prognosis and confounds these results. The equivocal results from these studies suggest that the necrosis that ensues from ischemia is more important than the tumoricidal effect of chemotherapeutic agents used.

Minor complications (post-embolization syndrome) are not uncommon but severe complications (biliary cirrhosis, abscess, and mortality) are rare [50,58]. This available data makes a case for using TAE/TACE in combination with other modalities (cytoreductive surgery) with a meaningful survival advantage, especially since TAE/TACE has the potential to convert a patient who is unresectable to resectable by decreasing tumor burden and increasing future liver remnant.

### 3.2. Transarterial Radioembolisation

TARE is the most recently developed embolization method that has been applied to patients with NETLM. Like TAE/TACE, it is most often used as a salvage therapy in patients with unresectable or borderline resectable lesions. Patients who are likely to respond well to TARE have minimal tumor vascular involvement and low histologic grade [65]. In a large, multi-institutional, single arm study of 148 patients who underwent TARE, they observed a complete or partial imaging response rate of 63% and a median OS of 70 months [66]. Unfortunately, this high median OS does not appear to have been replicated and subsequent studies have demonstrated a median OS ranging from 34–36 months (Table 3).

There is a paucity of data that compares the efficacy of TARE and TACE. In one retrospective uncontrolled comparison, TARE and TAE had similar one year survival rates [70]. In another study, TACE had a similar median OS and progression free survival (PFS) despite a significantly higher disease control rate than the TARE group (96% vs. 83%, *p* < 0.01) [73]. In a three-arm retrospective study (cTACE to DEB-TACE to TARE), cTACE had a higher five-year OS and hepatic PFS than DEB-TACE or TARE [71]. Additionally, DEB-TACE and TARE had similar five-year survival. Finally, in another retrospective three-arm study comparing TAE, TACE and TARE, there was no significant difference in median OS or PFS among the three groups, although the TACE group had a higher disease control rate [51]. Overall, retrospective studies have done little to clarify which modality is superior likely due to retrospective nature of the studies and heterogeneity of the study populations.

Although biochemical toxicities are reportedly higher with TARE, the rate of severe toxicities is similar among the three methods (8–11%) [51]. Given the paucity of literature that compares the three liver directed methods and the similarity in severe morbidity and outcomes, it appears that any of the embolotherapy methods could be used in patients with unresectable disease. However, in patients who are borderline resectable, TACE might be the superior method since it has better disease response rates that could convert patients who are borderline resectable to resectable. Results from a prospective randomized controlled trial such as the RETNET trial, which is randomizing patients into TAE, cTACE and DEB-TACE, are yet to clarify the superior embolization modality [74]. In the meantime, we await prospective data comparing TARE to conventional embolization.

## 4. Peptide Receptor Radionuclide Therapy

PRRT uses somatostatin analogues (SSA) that are labelled with a radioactive molecule by a chelator to deliver targeted radiotherapy and was initially reported to be used in humans in 1987 in Europe [75]. It was initially shown to stabilize disease in patients with advanced inoperable or end-stage neuroendocrine tumors and later proven to prolong survival, [76] with median OS ranging from 14–36 months (Table 4). Severe side effects of PRRT are uncommon and include gastrointestinal, nephrotoxicity, hepatotoxicity, and bone marrow complications (cytopenia (1–9%) and myelodysplastic syndrome (0.9–2%)) [77]. Imaging response to therapy has been associated with improved survival outcomes [78]. In one of the largest single arm cohort studies of 310 patients with GEP-NETs to determine efficacy of ^177^Lu-octreotate against NETLM, the authors found median PFS and OS of 33 months and 46 months [79], which were higher than had been previously reported in historic series [80].

Patient selection is important for successful treatment with PRRT. PRRT appears to be most beneficial in patients with well-differentiated tumors with a Ki67 < 20 (G1/G2) [81] and patients who have had primary tumor resection [89]. However, PRRT may also be used in a subset of patients with G3 tumors (Ki67 < 55%) with a reported median PFS and OS of 14 months and 29 months, respectively [86].

The approval of PRRT use in the USA is based on the NETTER-1 trial, a multicenter, prospective, randomized controlled trial that compares the efficacy and safety of ^177^Lu-Dotatate to long-acting repeatable (LAR) octreotide in G1/G2 GI-NETs [84]. Of the 221 patients included in the analysis, 83% had NETLM and the rate of prior surgical resection was similar in both groups. There was a 79% lower risk of disease progression to death in the treatment arm compared to the control group, and a higher median PFS that had not yet been reached in the treatment arm compared to 8.4 months in the control group. Although this study did not include any patients with PNETs it is still the highest quality evidence showing the superiority of PRRT to LAR octreotide in patients with NETLM. Until the NETTER-2 trial which includes PNET patients is published, the combined results from NETTER-1 and retrospective studies that have included PNET patients will be the basis for use of PRRT in patients with PNETs.

Results of the NETTER-1 trial have confirmed the superiority of PRRT to LAR which has fueled the debate about when PRRT should be used in the treatment algorithm. There is growing interest in use of PRRT in the neoadjuvant setting [85] however, sample sizes have been small. At our institution, a prospective pilot study is underway to determine the efficacy of PRRT in the neoadjuvant setting for patients with resectable G1/G2 tumors (ClinicalTrials.gov NCT04609592). The benefits of using PRRT in the neoadjuvant setting would need to be weighed against the adverse side effects of PRRT and potential for increasing interaction with other treatment modalities in the future such as radioembolization.

## 5. Conclusions

There are multiple treatment modalities for the management of pancreatic neuroendocrine metastases ranging from SSAs to surgical operations with varying levels of invasiveness, efficacy, and risk. Providers employ as many tools in their arsenal as possible to prolong survival and minimize adverse interactions of the different treatment modalities.

Surgery offers the greatest survival benefit for patients and provides the most significant symptom control. Unfortunately, a majority of patients are ineligible for surgical resection. It is for this group of patients that alternative treatment modalities such as embolization and PRRT are most beneficial. Patients with borderline resectable or unresectable liver metastases are well-suited to embolization as this modality could convert them to resectable disease while increasing the future liver remnant. Among the three embolotherapy modalities, current data supports TACE over band embolization and TARE (improved disease response rate); however, survival outcomes are similar. Bland embolization could still be employed if TACE is technically not feasible. PRRT is a relatively new systemic therapy which is particularly suitable for patients with multi-organ metastases and offers a similar survival benefit to the liver directed therapies. Additionally, it could be used in a subset of G3 tumors unlike the other therapies that are typically applied to patients with more favorable tumor biology. Currently, it is most commonly used in patients as salvage therapy and in patients who have already undergone surgical resection. The role of PRRT as it relates to surgery is evolving as new data regarding neoadjuvant use or use with concurrent systemic chemotherapy emerges.

A limitation of this review is that many studies included in the review are comprised of heterogeneous study populations. As a result, conclusions about outcomes of PNET patients are drawn from outcomes of GEP-NET patients although outcomes vary by site of primary tumor. Specifically, PNETs portend a poorer prognosis than other GEP-NETs. Furthermore, most studies included in this review are observational and retrospective, hence prone to selection bias, e.g., patients who undergo surgery are likely to be healthy enough to undergo an operation and also have favorable biology which contribute to prolonged survival. Nevertheless, these studies provide crucial data on which clinical decisions for rare diseases are based, despite the limitations.

## Figures and Tables

**Table 1:** Study outcomes of resection of primary tumor with or without hepatic metastasectomy of gastroenteropancreatic neuroendocrine tumors.

Author	Year	N	PNET (N)	Groups	Sx Resp (%)	BC Resp (%)	Morbidity (%)	Mortality (%)	Median OS (mo)	Median PFS (mo)	Five-Year Survival (%)
Que [15]	1995	74	≥23	-	90	-	24	2.7	-	-	-
Norton [16]	2003	16	2	-	-	100	-	0	32	-	82
Sarmiento [13]	2003	170	52	-	96	-	14	1.2	81	-	61
Givi [17]	2006	60	unk	Resected PT	-	-	-	-	159	56	81
		24	unk	Unresected PT	-	-	-	-	47	25	21
^#^ Kazanjian [18]	2006	70	70	-	-	-	-	0	-	-	89
Osborne [19]	2006	59	16	Embolization	91	-	-	-	24 *	-	-
		61	16	cytoreduction	93	-	2	1	43 *	-	-
^#^ Schurr [20]	2007	62	62	-	-	-	-	-	57	-	64
Nguyen [21]	2007	73	73	-	-	-	27	2.7	48	-	44
Chambers [22]	2008	66	unk	-	75	-	22	0	-	-	74
^#^ Hill [23]	2009	310	310	Resected	-	-	-	-	114	-	-
		417	417	unresected	-	-	-	-	35	-	-
Glazer [24]	2010	172	55	-	-	-	22	-	115	-	77
Mayo [25]	2010	339	134	-	-	-	-	-	125	-	74
Mayo [14]	2011	339	134	Surgery	-	-	-	-	123	-	74
		414	105	IAT	-	-	-	-	34	-	30
Cheung [26]	2014	12	6	-	-	-	25	-	53	-	-
Graff-Baker [27]	2014	52	unk	-	-	-	-	-	-	72	88
Birnbaum [28]	2015	91	91	isoPNET	-	-	21	5	-	-	87
		43	43	advPNET	-	-	19	2	-	-	66
Partelli [29]	2015	91	91	Resection	-	-	-	2	97	-	76
		75	75	No resection	-	-	-	-	36	-	36
Keutgen [30]	2016	303	303	Resection	-	-	-	-	65	-	-
		579	579	No resection	-	-	-	-	10	-	-
Maxwell [31]	2016	108	28	-	-	-	13	0	nr	38	-
Morgan [32]	2018	42	42	-	-	-	18	0	-	-	81
Feng [33]	2019	50	50	PT resected	-	-	-	-	12	-	-
		290	290	PT unresected	-	-	-	-	8	-	-
^#^ Scott [34]	2019	184	41	-	45	69	52	-	89	23	-
^#^ Tierney [35]	2019	460	460	PT resected	-	-	-	-	64	-	-
		5628	5628	PT unresected	-	-	-	-	14	-	-
^#^ Titan [36]	2020	99	99	-	-	-	-	2	-	-	91

* mean survival; ^#^ primary tumor resection only; PT, primary tumor; IAT, intra-arterial therapy; isoPNET, isolated pancreatic neuroendocrine tumor; advPNET, advanced pancreatic neuroendocrine tumor; N, number; Sx Resp, symptom response; BC Resp, biochemical response; PFS, progression free survival; OS, overall survival; nr, not reported; unk, unknown; PNET, pancreatic neuroendocrine tumor.

**Table 2:** Study outcomes of transarterial embolization (TAE) and transarterial chemoembolization (TACE) in patients with neuroendocrine liver metastases.

Author	Year	Tumor Type	*N*	PNET (*N*)	Rx	Sx resp (%)	BC resp (%)	Imaging Response (%)			Morbidity (%)	30-Day Mortality	Median PFS/TTP(mo)	Median OS (mo)	Five-Year Survival (%)
								CR+PR	SD	PD					
**Ruszniewski** [61]	1993	CarcinoidIslet cell	18 5	0 5	TACE	73 -	57 -	33 -	- 60	- -	- -	- -	- -	- -	- -
**Kress** [50]	2003	NET	26	9	TACE	-	-	8	54	19	35	8	NR	NR	48
**Gupta** [52]	2005	Carcinoid Islet cell	69 54	0 54	TAE/ TACE	- -	-	67 35	25 61	9 4	20 ^#^, 12 ^¶^	NR	23 16	34 23	27 14
**Osborne** [19]	2006	NET	59	16	TAE	91	-	-	-	-	-	0	-	24	-
**Strosberg** [57]	2006	NET	84	20	TAE	80	80	48	52	-	-	0	-	36	-
**Ho** [59]	2007	Carcinoid Islet cell	31 15	0 15	TAE/TACE	78 75	- -	23 18	32 45	23 9	10 ^¥^	4 ^¥^	23 16	42 44	32 35
**Bloomston** [53]	2007	Carcinoid	122	26	TACE	92	80	82	12	6	23	5	-	33	-
**Ruutiainen** [60]	2007	Carcinoid NET	44 23	≥14	TACE TAE	92 93	- -	66 50	22 38	12 13	25 22	1 ^¥^	55 10	44 39	49 39
**Pitt** [58]	2008	NET	49 51	44	TACE TAE	86 83	- -	- -	- -	- -	7 2	0.8 2	- -	26 26	19 13
**Dong** [56]	2011	NET	50	unk	TACE	-	-	62	24	14	0	-	-	65	36
**Mayo** [14]	2011	Carcinoid/ PNET	414	105	TACE/TAE/ TARE	-	-	6	41	33	48	2	-	34	30
**Maire** [62]	2012	NET	12 14	0	TACE TAE	- -	67 82	100 92		0 8	-	-	0 ^¥^	-	-
**Bhagat** [63]	2013	NET	13	5	TACE	-	-	-	-	-	54	0	-	-	-
**Fiore** [64]	2014	NET	17 13	6 6	TAE TACE	- -	- -	- -	- -	- -	41 61	- -	60 36	- -	- -
**Dhir** [55]	2017	NET	91	22	TACE	54	-	43	38	19	10	2	18	44	41

^¥^ entire cohort; ^#^ TACE; ^¶^ TAE; *N*, number; CR, complete response; PR, partial response; SD, stable disease; PD, progressive disease; PFS, progression free survival; TTP, time to progression, OS, overall survival; nr, not reported; TARE, transarterial radioembolization; PNET, pancreatic neuroendocrine tumor; NET, neuroendocrine tumor; yr, year; unk, unknown.

**Table 3:** Review of literature on transarterial radioembolization (TARE) of neuroendocrine tumor liver metastases.

Author	Year	*N*	PNET (*N*)	Rx	Sx Resp (%)	Imaging			Median PFS (mo)	Median OS (mo)	One-year Survival (%)	Five-Year Survival (%)	30-Day Morbidity (mo)	30-Day Mortality (mo)
						CR+PR (%)	SD (%)	PD (%)						
Rhee [67]	2008	22	7	TARE Glass	-	54	38	8	-	22	-	-	-	5 ^¥^
		20	4	TARE Resin	-	50	44	6	-	28	-	-	-	-
Kennedy [66]	2008	148	28	TARE	-	63	23	5	-	70	-	-	33	-
Cao [65]	2010	51	14	TARE	-	39	27	33	-	36	-	-	-	2
Memon [68]	2012	40	9	TARE	84	64	33	4	-	34	73	-	-	-
Paprottka [69]	2012	42	9	TARE	95	23	75	3	-	-	-	-	-	-
Gebhard [70]	2013	17	unk	TARE	-	-	-	-	-	-	68		12	-
		29		TAE	-	-	-	-	-	-	82		3	-
Chen [51]	2017	50	23	TACE	-	-	-	-	8	33	82	-	-	-
		64	26	TARE	-	-	-	-	16	48	79	-	-	-
		41	22	TAE	-	-	-	-	15	-	90	-	-	-
Do Minh [71]	2017	122	44	cTACE	-	3	92	4	-	34	81	28	85	-
		26	10	DEB-TACE	-	4	92	4	-	22	73	10	89	-
		44	13	TARE	-	0	89	11	-	24	71	19	84	-
Tomozawa [72]	2018	93	27	TARE	-	25	67	8	-	-	-	-	-	-
Egger [73]	2020	51	16	TARE	-	24	59	17	16	36	-	35	14	2
		197	46	TACE	-	30	66	4	20	50	-	42	23	3

^¥^ for entire cohort; *N*, number of patients; Rx, treatment; Sx Resp, symptom response; TAE, transarterial embolization; TACE, transarterial chemoembolization; cTACE, conventional transarterial chemoembolization; DEB-TACE, drug-eluting bead transarterial chemoembolization; CR, complete response; PR, partial response; SD, stable disease; PD, progressive disease; PFS, progression free survival; OS, overall survival; mo, months; yr, year; PNET, pancreatic neuroendocrine tumor; unk, unknown.

**Table 4:** Studies of use of peptide receptor radionuclide therapy (PRRT) in neuroendocrine liver metastases.

Author	Year	Tumor Type	*N*	PNET (*N*)	Rx Arm	Sx Resp (%)	BC Resp (%)	Imaging Response (%)			Morbidity (%)	Mortality (%)	Median PFS/TTP (mo)	Median OS/DSS (mo)	Five-Year Survival (%)
								CR+PR	SD	PD					
Valkema [80]	2006	GEPNET	58	≥14	-	58		9	62	29	20	8	14/-	37/-	-
Kwekkeboom [79]	2008	GEPNET	310	≥79	-	-	-	29	51	20	0.4	-	33/-	46/-	-
Bodei [81]	2011	NET	51	14	-	-	-	33	53	18	-	27 ^#^	-/36	nr/-	-
Ezziddin [82]	2011	GEPNET	81	37	-	-	-	38 *	46 *	16 *	-	-	-/-	-/-	-
Ezzidddin [83]	2011	GEPNET	42	12	-	100 ^¥^	-	72	38	12	-	0	35/-	51/-	-
Bertani [78]	2016	PNET	94	94	-	-	-	26	42	32	-	-	36/-	76/-	-
Strosberg [84]	2017	GI NET	111	0	-	-	-	18	-	-	0.9–9	-	nr/-	nr/-	-
Partelli [85]	2018	PNET	23	23	PRRT first	-	-	70	22	8	4	-	52/-	-/nr	-
		PNET	23	23	Upfront surgery	-	-	-	-	-	-	-	37/-	-/nr	-
Carlsen [86]	2019	GEPNET	149	89	-	-	-	42	38	20	17	-	14/-	29/-	-
Satapathy [87]	2020	NET	45	14	-	75	50	30	55	15	2–4	-	48/-	84/-	-
Sistani [88]	2020	NET	47	13	-	-	-	32	53	15	-	-	36/-	nr/-	-
Kaemmerer [40]	2021	GEPNET	486	148	PT resection	-	-	-	-	-	-	-	18/-	134/-	71
		GEPNET	403	187	No PT resection	-	-	-	-	-	-	-	14/-	67/-	42

* SWOG tumor response criteria; ^#^ died from disease progression; ^¥^ denominator is patients with pain from osseous metastases; GEPNET, gastroenteropancreatic neuroendocrine tumor; NET, neuroendocrine tumor; PNET, pancreatic neuroendocrine tumor; GI NET, gastrointestinal neuroendocrine tumor; N, number of patients; Rx arm, treatment arm; Sx Resp, symptomatic response; BC Resp, biochemical response; CR, complete response; PR, partial response; SD, stable disease; PD, progressive disease; PFS, progression free survival; TTP, time to progression; OS, overall survival; DDS, disease specific survival; mo, months; nr, not reached; yr, year.
